# Integrating Patient Data Into Skin Cancer Classification Using Convolutional Neural Networks: Systematic Review

**DOI:** 10.2196/20708

**Published:** 2021-07-02

**Authors:** Julia Höhn, Achim Hekler, Eva Krieghoff-Henning, Jakob Nikolas Kather, Jochen Sven Utikal, Friedegund Meier, Frank Friedrich Gellrich, Axel Hauschild, Lars French, Justin Gabriel Schlager, Kamran Ghoreschi, Tabea Wilhelm, Heinz Kutzner, Markus Heppt, Sebastian Haferkamp, Wiebke Sondermann, Dirk Schadendorf, Bastian Schilling, Roman C Maron, Max Schmitt, Tanja Jutzi, Stefan Fröhling, Daniel B Lipka, Titus Josef Brinker

**Affiliations:** 1 Digital Biomarkers for Oncology Group (DBO) National Center for Tumor Diseases (NCT) German Cancer Research Center (DKFZ) Heidelberg Germany; 2 Department of Medicine III RWTH University Hospital Aachen Aachen Germany; 3 National Center for Tumor Diseases (NCT) German Cancer Research Center (DKFZ) Heidelberg Germany; 4 Department of Dermatology University Hospital of Mannheim Mannheim Germany; 5 Skin Cancer Unit German Cancer Research Center Heidelberg Germany; 6 Skin Cancer Center at the University Cancer Centre and National Center for Tumor Diseases Dresden Department of Dermatology University Hospital Carl Gustav Carus, Technische Universität Dresden Dresden Germany; 7 Department of Dermatology University Hospital of Kiel Kiel Germany; 8 Department of Dermatology and Allergology Ludwig Maximilian University of Munich Munich Germany; 9 Department of Dermatology, Venereology and Allergology Charité – Universitätsmedizin Berlin Berlin Germany; 10 Dermatopathology Laboratory Friedrichshafen Germany; 11 Department of Dermatology University Hospital Erlangen Erlangen Germany; 12 Department of Dermatology University Hospital of Regensburg Regensburg Germany; 13 Department of Dermatology University Hospital Essen Essen Germany; 14 Department of Dermatology University Hospital Würzburg Würzburg Germany; 15 Translational Cancer Epigenomics, Division of Translational Medical Oncology German Cancer Research Center Heidelberg Germany; 16 Faculty of Medicine, Medical Center Otto-von-Guericke-University Magdeburg Germany

**Keywords:** skin cancer classification, convolutional neural networks, patient data

## Abstract

**Background:**

Recent years have been witnessing a substantial improvement in the accuracy of skin cancer classification using convolutional neural networks (CNNs). CNNs perform on par with or better than dermatologists with respect to the classification tasks of single images. However, in clinical practice, dermatologists also use other patient data beyond the visual aspects present in a digitized image, further increasing their diagnostic accuracy. Several pilot studies have recently investigated the effects of integrating different subtypes of patient data into CNN-based skin cancer classifiers.

**Objective:**

This systematic review focuses on the current research investigating the impact of merging information from image features and patient data on the performance of CNN-based skin cancer image classification. This study aims to explore the potential in this field of research by evaluating the types of patient data used, the ways in which the nonimage data are encoded and merged with the image features, and the impact of the integration on the classifier performance.

**Methods:**

Google Scholar, PubMed, MEDLINE, and ScienceDirect were screened for peer-reviewed studies published in English that dealt with the integration of patient data within a CNN-based skin cancer classification. The search terms *skin cancer classification*, *convolutional neural network(s)*, *deep learning*, *lesions*, *melanoma*, *metadata*, *clinical information*, and *patient data* were combined.

**Results:**

A total of 11 publications fulfilled the inclusion criteria. All of them reported an overall improvement in different skin lesion classification tasks with patient data integration. The most commonly used patient data were age, sex, and lesion location. The patient data were mostly one-hot encoded. There were differences in the complexity that the encoded patient data were processed with regarding deep learning methods before and after fusing them with the image features for a combined classifier.

**Conclusions:**

This study indicates the potential benefits of integrating patient data into CNN-based diagnostic algorithms. However, how exactly the individual patient data enhance classification performance, especially in the case of multiclass classification problems, is still unclear. Moreover, a substantial fraction of patient data used by dermatologists remains to be analyzed in the context of CNN-based skin cancer classification. Further exploratory analyses in this promising field may optimize patient data integration into CNN-based skin cancer diagnostics for patients’ benefits.

## Introduction

### Background

The incidence of skin cancer has been increasing throughout the world, resulting in substantial health and economic burdens [1]. Early detection increases the possibility of curing all types of skin cancers. However, distinguishing benign skin lesions from malignant skin lesions is challenging, even for experienced clinicians [2]. Over the past few years, different digital approaches have been proposed to assist in the detection of skin cancer [3,4]. Convolutional neural networks (CNNs) are the most successful systems for handling image classification problems [5]. Recent publications have reported CNNs that support [6] and outperform [7-9] dermatologists in challenging binary melanoma detection and multiclass skin cancer classification when only taking single images of the skin lesion as input.

**Figure 1:**
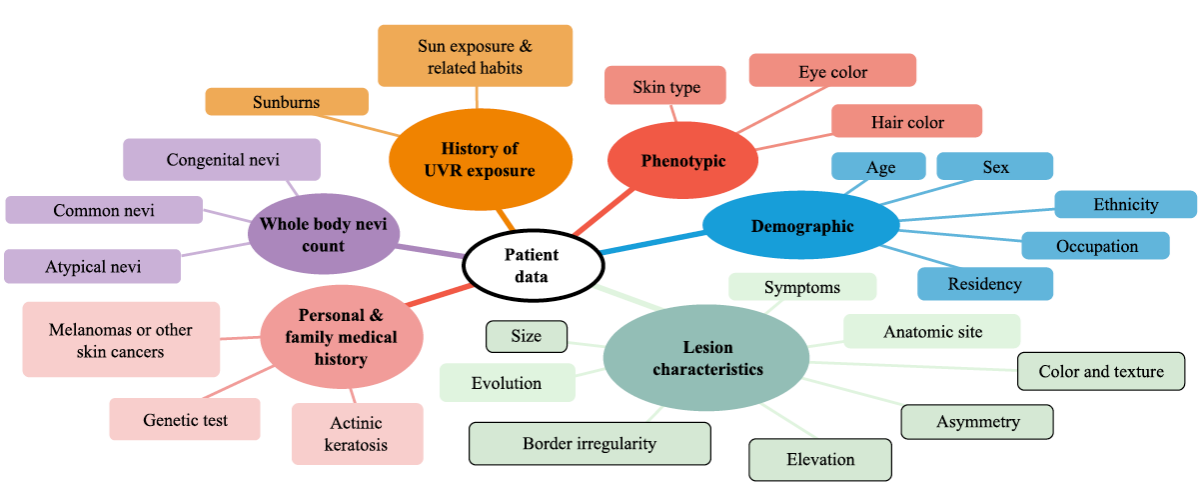
An overview of patient data considered by dermatologists while diagnosing skin lesions. The framed characteristics in the figure illustrate the fraction of patient data that can potentially be recognized by convolutional neural networks from a single image input. UVR: ultraviolet radiation.

However, single-image classification does not reflect the clinical reality. In fact, dermatologists’ diagnoses are based on both the visual inspection of a single image and the integration of information from various sources. Figure 1 shows the different types of information that dermatologists may collect from their patients, including information on known risk factors for skin cancer. The corresponding references can be found in Multimedia Appendix 1 [10-32]. The complexity of this figure illustrates the diversity of patient data that can be included in the diagnosis. Roffman et al [33] and Wang et al [34] presented reasonably accurate skin cancer predictions using deep learning methods based exclusively on patient nonimage information. Haenssle et al [35] showed that dermatologists perform somewhat better in a dichotomous skin lesion classification task (benign vs malignant or premalignant) when they were provided with clinical images and textual case information, such as the patient’s age, sex, and lesion location, in addition to a dermoscopic image. This raises the question of whether a combination of CNN-based image analysis and patient data might also increase the accuracy of the classifier. A combination of image features and patient data has become a topic of the International Skin Imaging Collaboration challenge in 2019, where a large repository of dermoscopic images including patient data was offered for clinical training and technical research toward automated algorithmic analysis [36].

### Objective

This review presents the status quo of CNN-based skin lesion classification using image input and patient data. The included studies were analyzed with respect to the amount and type of patient data used for integration, the encoding and fusing techniques, and the reported results. The review also discusses the heterogeneity of the studies that have been conducted so far and points out the potential and challenges of such combined classifiers that should be addressed in the future.

## Methods

### Search Strategy

Google Scholar, PubMed, MEDLINE, and ScienceDirect were searched for peer-reviewed publications, restricted to human research published in English. The search terms *skin cancer classification*, *convolutional neural network(s)*, *deep learning*, *lesions*, *melanoma*, *metadata*, *clinical information*, and *patient data* were combined.

### Study Selection

This review only includes skin lesion classification studies using CNNs that consider both image and patient data. It must be noted that there are a few studies that investigated the incorporation of visual and nonvisual information on skin cancer classification, but did not obtain visual features using deep learning techniques, for example, the studies by Binder et al [37], Alcon et al [38], Cheng et al [39], Liu et al [40], and Rubegni et al [41]. This review exclusively focuses on integrating patient data with the state-of-the-art CNN-based feature extractors. Therefore, the abovementioned studies were not considered in this review.

### Study Analysis

The objective of this review is to update practitioners on the status quo approaches toward patient data incorporation into CNN-based skin lesion diagnostics regarding all relevant practical aspects.

#### Type and Amount of Patient Data

The goal is to achieve better performance of the CNN-based classifier by integrating new information that cannot be extracted from a digitized image. Various types of patient data have been shown to assist dermatologists. *Key question:* Which and how many different types of patient data have been tested for CNN-based classification?

#### Encoding and Fusing Techniques

A CNN-based classifier extracts various visual features from a digitized image as the basis for its diagnosis. Patient data are nonimage data and are mostly provided as numbers or strings in tables. The patient data can be classified in a dichotomous fashion (presence of the feature: yes or no), fall into several discrete categories (eg, Fitzpatrick skin type), or be continuous (eg, patient age). This may require different, carefully chosen encoding and fusing techniques. Moreover, the weight attributed to patient data in comparison with image features can strongly influence how the different features contribute toward the decision making of the system. *Key questions:* What are the encoding and fusing techniques applied in the studies? Do the studies focus on the quantitative relationship between image and nonimage features?

#### Reported Study Results

This review aims to summarize the recent findings regarding the impact of patient data on the performance of CNN-based classifiers. *Key questions:* What is the classification task? Is it a binary or multiclass problem? Which skin lesions should be distinguished? How is the influence of individual and/or combined patient data documented? In the case of multiclass classification, is the impact also shown for each single class of skin lesion individually?

### Applied Performance Metrics

The included publications reported different statistical metrics as the study end points. If the classes in the test set are approximately equally distributed, then accuracy is a frequently used performance metric, where the total number of correctly predicted samples is divided by the total number of samples in the test set. In binary classification problems with a positive and a negative class, sensitivity and specificity are further common study end points, especially if there is an imbalance between the samples of both classes. Sensitivity was determined only on the basis of the actual positive samples in the test set. It is calculated by counting the correctly classified positive samples by the total number of positive samples. In contrast, specificity was determined based on actual negative samples in the test set. Here, the correctly classified negative samples were divided by the total number of negative samples. While using a CNN, the sensitivity and specificity depend on the selected cutoff value. If the output of the neural network is greater than the cutoff value, the input is assigned to the positive class, and if it is below that value, then the input is assigned to the negative class. Thus, this value represents a central parameter for the trade-off between sensitivity and specificity. A decrease in the threshold value leads to an increase in the sensitivity with a simultaneous decrease in specificity and vice versa. The dependence of the cutoff value of the specificity and sensitivity of the two metrics is shown in the receiver operating characteristic curve. Here, the sensitivity is plotted against the false-positive rate (1−specificity) in a diagram for each possible cutoff value. The area under the receiver operating characteristic curve was used as an integral performance measure for the algorithms.

## Results

### Classification Tasks

A total of 11 publications fulfilling the inclusion criteria are summarized in Table 1. The studies were very heterogeneous with respect to CNN architecture, classification task, including image and patient data, data augmentation (if reported), and fusion techniques, rendering a meaningful direct comparison very difficult. A total of 5 studies dealt with binary classifications with the end point of either dichotomous melanoma or basal cell carcinoma (BCC) classification or with the end point to distinguish malignant from benign lesions in general. The remaining 6 studies were classified between 5 and 8 different skin diseases or lesions. As malignant lesions, melanoma (6 studies), BCC (6 studies), and squamous cell carcinoma (SCC; 3 studies) were included. In addition, these studies differentiated among the following benign lesions: melanocytic nevus (NV; 6 studies), benign keratosis-like lesions (BKLs; 4 studies), dermatofibroma (3 studies), vascular lesions (VASCs; 3 studies), actinic keratosis (AK; 2 studies), and seborrheic keratosis (SK; 2 studies). Moreover, merged groups comprising AK and intraepithelial carcinoma or Bowen disease (2 studies) and dermatofibroma, lentigo, melanosis, miscellaneous, and VASCs (1 study) were used in some of the studies.

**Table 1:** Summary table.

Study	Patient data types	Result (without/with)	Classification task	CNN^a^ architecture	Data set	Samples, n
Bonechi et al [42]	4 types: age, sex, location, and presence of melanocytic cells	Accuracy: 0.8344/0.8834	Binary: benign or malignant (MEL^b^, BCC^c^, SCC^d^)	ResNet50	ISIC^e^	5405
Chin et al [43]	5 types: age; sex; size; how long it existed; changes in size, color, or shape including bleeding and itching	Accuracy: 0.84/0.92	Binary: low risk or high risk for MEL	DenseNet121	Own	5289
Gonzalez-Diaz [44]	2 types: age and sex	Accuracy: 0.848/0.859	Binary: MEL yes or no	ResNet50	2017 ISBI^f^ challenge+interactive atlas of dermoscopy [45]+ISIC	6302
Gessert et al [46]	3 types: age, sex, and location	Sensitivity: 0.725/0.742; specificity: data not available	8 classes: MEL, NV^g^, BCC, AK^h^, BKL^i^, DF^j^, VASC^k^, SCC	EfficientNets	ISIC (HAM10000 [47], BCN_2000 [48], MSK [49])+7-point data set [50]	27,665
Kawahara et al [50]	3 types: sex, location, and elevation	Sensitivity: 0.527/0.604; specificity: 0.902/0.910	5 classes: MEL, BCC, NV, MISC^l^, SK^m^	Inception V3	7-point data set	808
Kharazmi et al [51]	5 types: age, sex, location, size, and elevation	Accuracy: 0.847/0.911	Binary: BCC yes or no	Convolutional filters of learned kernel weights from a sparse autoencoder	Own	1199
Li et al [52]	3 types: age, sex, and location	Sensitivity: 0.8544/0.8764; specificity: data not available	7 classes: NV, MEL, BKL, BCC, AKIEC^n^, VASC, DF	SENet154	ISIC 2018 data set	10,015
Pacheco and Krohling [53]	8 types: age, location, lesion itches, bleeds or has bled, pain, recently increased, changed its pattern, and elevation	Accuracy: 0.671/0.788	6 Classes: BCC, SCC, AK, SK, MEL, NV	ResNet50	Own	1612
Ruiz-Castilla et al [54]	3 types: age, sex, and size	Accuracy: 0.61/0.85	Binary: MEL yes or no	Shallow network with 2 convolutional layers	ISIC	300
Sriwong et al [55]	3 types: age, sex, and location	Accuracy: 0.7929/0.8039	7 classes: AKIEC, BCC, BKL, DF, MEL, NV, VASC	AlexNet	HAM10000	16,720
Yap et al [56]	3 types: age, sex, and location	Mean average precision: 0.726/0.729; Accuracy: 0.721/0.720	5 classes: BCC, SCC, MEL, BKL, NV	ResNet50	ILSVRC^o^ 2015 [57]+own	2917 (only testing)

^a^CNN: convolutional neural network (most of the studies had the goal of investigating the usefulness of the presented fusion technique independently of the convolutional neural network architecture and, therefore, often showed the performance of the fusion with multiple architectures; we included only the best-performing architecture).

^b^MEL: melanoma.

^c^BCC: basal cell carcinoma.

^d^SCC: squamous cell carcinoma.

^e^ISIC: International Skin Imaging Collaboration.

^f^ISBI: International Symposium on Biomedical Imaging [49].

^g^NV: melanocytic nevus.

^h^AK: actinic keratosis.

^i^BKL: benign keratosis-like lesion.

^j^DF: dermatofibroma.

^k^VASC: vascular lesion.

^l^MISC: summary of dermatofibroma, lentigo, melanosis, miscellaneous, and vascular lesion.

^m^SK: seborrheic keratosis.

^n^AKIEC: actinic keratosis and intraepithelial carcinoma.

^o^ILSVRC: ImageNet Large Scale Visual Recognition Challenge.

### Types and Amount of Patient Data

Most of the studies included three types of patient data (7/11, 64%). Compared with the diversity of potentially useful patient data illustrated in Figure 1, only a few types of patient data were considered. The most commonly included types of data were patient’s age and sex (studies: 10/11, 91%). Only Kawahara et al [50] and Pacheco and Krohling [53] did not consider age and sex, respectively. The third most commonly considered feature was lesion location (studies: 8/11, 73%). Elevation and lesion size were considered in 27% (3/11) of studies. Chin et al [43] and Pacheco and Krohling [53] included statements about symptoms such as itching, bleeding or pain. In addition, they tracked the lesion’s evolution by documenting whether the lesion increased in size or changed its shape. Furthermore, Bonechi et al [42] considered the presence of melanocytic cells as an additional potentially relevant feature.

### Encoding

The means of choice to encode the patient data was one-hot encoding in most cases. One-hot encoding is one way to encode several discrete classes with a string of bits, where exactly one value in the string of bits encoding one class is assigned 1 and all others are assigned 0 (eg, *melanoma*=010; *BCC*=100; *NV*=001). Different techniques were used for continuous parameters such as the patient’s age. One-hot encoding is only possible after discretizing the continuous range, which was performed by Bonechi et al [42], who divided the age ranging from 0 to 95 in the sections of 5 years. Gessert et al [46] tested numerical against one-hot encoding and found the former to be superior. Li et al [52] normalized the age in the range between 0 and 1 and represented its information using only one value.

As patient data are rarely documented in a standardized way, dealing with missing values is an essential skill that requires the algorithm to be proficient. However, only 18% (2/11) of publications went into detail on how they dealt with missing values. Gessert et al [46] suggested a negative fixed value for missing data, whereas Li et al [52] used the more common approach to fill in missing values with average values for continuous data and the most frequent values for discrete patient data.

### Fusing Technique

**Figure 2:**
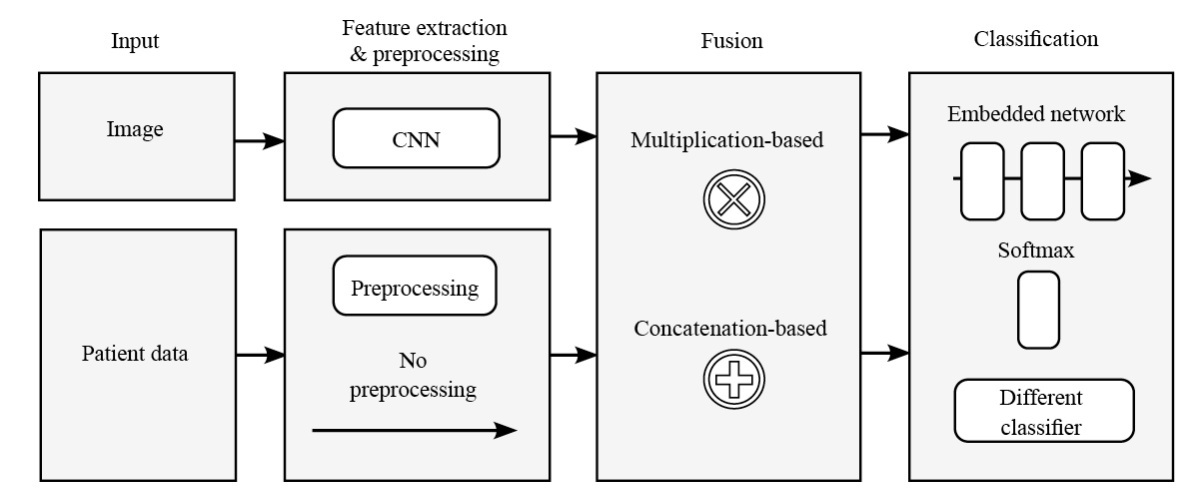
Overview of the different fusing techniques in the main function blocks of the combined classifier. CNN: convolutional neural network.

Figure 2 illustrates the main function blocks in which the studies vary with respect to the fusing techniques.

The fusing techniques differ in the way they actively weigh the image and patient data. In 82% (9/11) of studies, a concatenation-based fusion was applied, that is, the feature vector extracted from the images was enlarged by attaching the encoded patient data. In this case, weighting is achieved by defining the ratio between the number of features originating from the image and the patient data input. Common CNN architectures extract 1024, 2048, or even more features from the image input. In most studies, the authors decided to reduce the image features before concatenating them with patient data. In only 27% (3/11) of studies, the authors provided sufficient information on this point and revealed a considerable variance in the ratio of image features to patient data: 112 to 28 [53], 128 to 80 [42], and 2048 or 2×2048 to 11 [56]. However, reducing the image features should be done with care, as it is accompanied by a loss of information. Only Pacheco and Krohling [53] reported the effect of changing the ratio and proved its strong influence on the classification performance. A totally different weighting approach was introduced by Li et al [52]. This approach used a multiplication-based fusion. Inspired by the squeeze-and-excitation operation of a SENet network [58], the authors used patient data to control the importance of each image feature channel at the last convolutional layer. Thus, the network was able to focus on specific parts of the image feature based on patient data. The authors determined the multiplication-based fusion to be superior to the concatenation-based approach in multiple network architectures.

In addition, the studies vary in the extent to which deep learning methods were applied to the patient data before fusing or on the combined feature vector after fusing it with the image data. Sriwong et al [55] applied no further deep learning methods but used a separate support vector machine for classification, which received the image features extracted by the CNN and the encoded patient data as input. Gonzalez-Diaz [44] used a separate support vector machine for the patient data to generate a probabilistic output, which was factorized with the output of the CNN-based classifier to provide the final diagnosis of the system. Because of the end-to-end training of a neural network, a direct fusion within the CNN architecture was used in most studies. Kharazmi et al [51] simply added patient data before the last classifying softmax layer. More complex deep learning methods were applied by Gessert et al [46] before concatenation or using an embedded network comprising multiple fully connected layers after concatenation as presented by Pacheco and Krohling [53] and Yap et al [56].

### Reported Study Results

As summarized in Table 1, all but one study reported a considerable improvement in the classification performance when patient data were used in addition to image analysis. However, the authors consistently emphasized that patient data are only a support source and the image features clearly provide the main evidence [53,56]. Yap et al [56], who considered the features of age, sex, and lesion location for a multiclass problem (BCC, SCC, melanoma, BKL, NV), concluded in the discussion that their incorporation of patient data showed only a slight but not significant improvement in accuracy and recommended testing different features, such as nevus count, proportion of atypical nevi, and history of melanoma.

Although 5 studies reported results for binary classification tasks, 55% (6/11) of studies dealt with a multiclass classification problem, distinguishing between up to 8 different skin diseases, and revealed insights on how the use of patient data influences the classification performance for an individual type of skin lesion. Table 2 shows which individual classifications benefitted from the integration of patient data and whether this was achieved at the expense of others. Among the 6 studies, Gessert et al [46], Sriwong et al [55], and Li et al [52] dealt with comparable classification tasks and used the same patient data (age, sex, and location). All 3 studies identified improvements in the classification of BKL and dermatofibroma. Li et al [52] even listed an absolute increase in the sensitivity for dermatofibroma of approximately 20% (from 63.56% to 84.55%). It must be stated critically that the authors failed to mention the corresponding specificity, which makes it difficult to draw reliable conclusions. Furthermore, Table 2 shows that the improvements may go along with the degradation of classification performance for other lesion types [52,55] or that the improvement of sensitivity for one class may be paralleled by a decrease in specificity, as shown clearly by the results of Gessert et al [46]. In contrast, Kawahara et al [50] and Pacheco and Krohling [53] used different patient data (eg, elevation of the lesion) and reported an increase in sensitivity and specificity in almost all classes. Unfortunately, a deeper insight into the study results of Yap et al [56] was not possible because the confusion matrix, including the classification performance of the single-lesion types, was not legible.

**Table 2:** Influence of included patient data on the classification performance of the single skin diseases or lesions^a^.

Study, patient data, and metric		Skin disease										
		MEL^b^	NV^c^	BCC^d^	SCC^e^	AK^f^	AKIEC^g^	BKL^h^	DF^i^	VASC^j^	MISC^k^	SK^l^
**Gessert et al [46]: age, sex, location**												
	AUC^m^	+^n^	(+/−)^o^	−^p^	−	+	X^q^	+	+	−	X	X
	Sensitivity	−	−	−	−	−	X	−	−	−	X	X
	Specificity	+	+	+	+	+	X	+	+	+	X	X
**Sriwong et al [55]: age, sex, location**												
	Sensitivity	+	−	+	X	X	−	+	+	−	X	X
	Specificity	−	+	−	X	X	+	+	+/−	+	X	X
**Li et al [52]: age, sex, location**												
	Sensitivity	−	−	+	X	X	−	+	+	+	X	X
**Kawahara et al [50]: sex, location, elevation**												
	Sensitivity	+	+	+	X	X	X	X	X	X	+	+
	Specificity	+	+	+/−	X	X	X	X	X	X	+	+
**Pacheco and Krohling [53]: age, location, itches, bleeds, pain, increased, changed, elevation**												
	Sensitivity	+	+	+	+	+	X	X	X	X	X	+
	Specificity	+	+	+	−	+	X	X	X	X	X	+

^a^The study of Yap et al [56] is excluded because the confusion matrix was not legible. It must be noticed that there are some combinations where the outcome deteriorates by including patient data.

^b^MEL: melanoma.

^c^NV: Melanocytic nevus.

^d^BCC: basal cell carcinoma.

^e^SCC: squamous cell carcinoma.

^f^AK: Actinic keratosis.

^g^AKIEC: actinic keratosis and intraepithelial carcinoma.

^h^BKL: benign keratosis-like lesion.

^i^DF: dermatofibroma.

^j^VASC: vascular lesion.

^k^MISC: miscellaneous and vascular lesion.

^l^SK: seborrheic keratosis.

^m^AUC: area under the curve.

^n^Indicates improvement compared with classification performance without patient data.

^o^Indicates no change compared with classification performance without patient data.

^p^Indicates degradation compared with classification performance without patient data.

^q^This implies that the lesion type was not considered in the classification task of the study.

In total, 36% (4/11) of studies analyzed the influence of the used patient data on the classification performance in a more differentiated way. They showed the impact of either individual patient data or special combinations of patient data on classification performance, thereby providing a more detailed insight into the contribution of individual patient data.

As the only ones, Pacheco and Krohling [53] performed an exploratory analysis of the patient data within the used data set before observing the classification of the CNN. The authors considered eight types of patient data (age, location, lesion itches, lesion bleeds, lesion hurts [“pain”], recent increase in size, changed shape or pattern, and elevation) and six different types of skin lesions (BCC, SCC, AK, SK, melanoma, and NV). The exploratory analysis suggested that the patient data parameters such as “bleeding” and “pain” were suitable to differentiate between pigmented (NV, melanoma, and SK) and nonpigmented lesions (AK, BCC, and SCC), whereas the patient data parameters such as “changed its pattern” and “elevation” helped to identify melanomas. As “pain” was always denied in the case of AK, this feature seemed to be a promising discriminator. The analyzed patient data for SCC and BCC were very similar; therefore, no improvement was expected for these 2 skin diseases because of the integration of patient data. The classification results confirmed the exploratory analysis because the classifications of AK, melanoma, NV, and SK improved when patient data were incorporated into the classifiers, whereas the performance for BCC and SCC remained almost the same.

Li et al [52] considered three types of patient data (age, sex, and lesion location) and 7 skin lesion types (NV, melanoma, BKL, BCC, AK and intraepithelial carcinoma, VASC, and dermatofibroma). The study showed the overall classification performance for all possible combinations of patient data. The integration of parameter “location” resulted in the best classification performance, individually. The combination of patient data parameters of “age” and “location” provided the best result overall, whereas the parameter “sex” decreased performance upon integration. The authors concluded that the rare diseases of VASC and dermatofibroma are more location specific, whereas none of the skin diseases in question occur preferentially in men or women. Therefore, the authors recommended the use of “location” and the avoidance of “sex” in the combined classifier.

Sriwong et al [55] addressed the same problem as that of Li et al [52]. However, their study only analyzed some combinations of patient data (age, age+sex, and age+sex+location). The best overall result was achieved by incorporating the combination of “age,” “sex,” and “location.” Contrary to Li et al [52], the study yielded the largest improvement for the feature “age.” Although adding “sex” did not show a considerable improvement, additionally adding “location” increased the performance slightly. The authors stated that the information of “sex” and “location” is more powerful when used in combination, thereby confirming statements in related studies [40,59].

Bonechi et al [42] considered four types of patient data individually (age, sex, location, and presence of melanocytic cells) for a binary classification (malignant yes or no). Unfortunately, the analysis results have not been reported in detail, but the authors reported the parameter “presence of melanocytic cells” to be the most informative.

## Discussion

### Principal Findings

Although the main evidence for a good diagnosis is still provided by the image input, all 11 publications indicate a possible benefit of integrating patient data in CNN classifiers, as illustrated in Table 1. This corresponds with the results of other approaches that combine visual and nonvisual features for skin lesion classification [37-41], thereby suggesting it as a promising avenue of research. However, publication bias favoring studies with positive results cannot be excluded.

One focus of further research into combined CNN-based classifiers should be to render its classification process transparent, easy to understand, and applicable in a clinical setting. The 11 studies published so far have dealt with these aspects only marginally. Therefore, these issues need to be addressed in future studies to reliably reveal the potential of integrating patient data.

### Reproducibility, Comparability, and Generalization

No objective benchmarks exist in the field of integrating patient data into CNN-based classifiers. The heterogeneity of the studies conducted so far is substantial. This applies to the number and types of skin diseases or lesions to be classified, databases and data augmentation, CNN architectures, patient data, and fusion techniques. These aspects have a great influence on the way that the algorithm learns to diagnose the lesions in question and render it very difficult to reproduce and compare the approaches and results externally and independently. A way to solve this would be the more extensive use of external and publicly available data sets to objectively optimize the classification accuracy in an experimental setting. This needs to be done systematically in preparation for clinical trials that will be required to prove the algorithm’s generalizability and applicability in the clinic. In addition, the best way to handle missing data needs to be addressed.

### Transparency and Explainability

All presented studies lack an investigation of the impact of patient data individually and in combination on single-lesion classes. Both the fusion method and weight attributed to the patient data in addition to the biological significance itself may substantially influence the classification results. Further research should be dedicated to explaining the mechanisms by which the incorporation of these factors contributes to the decision making of the CNN-based combined classifier to render the results more transparent.

### Call for Extensive Exploration Analysis

As shown in Figure 1, a diversity of patient data has been shown to be useful in a clinical setting and could be considered for diagnosis by CNN-based classifiers as well. So far, researchers have mostly used patient data that are readily available and/or routinely recorded, such as age, sex, and lesion location. However, readily available factors may not be the best choice. For instance, sex was included in 91% (10/11) of studies, but was stated to be of minimal benefit for the classification task if investigated in detail. Regarding the results of studies considering patient data besides these three factors, the results indicate that the integration of other patient data may be more promising [39,42,53]. Studies analyzing the risk factors for skin cancer so far demonstrated that patient data can be helpful in distinguishing skin lesions in binary classification tasks. Corresponding studies are available for differentiating between melanoma and nonmelanoma skin cancer [10,60] and for distinguishing between BCC and SCC [61]. Patient data, such as the skin type (I, II, III vs IV), the count of atypical nevi (>4 vs none) and common nevi (>100 vs 0-4) are well-established criteria for melanoma risk [11,12]. To our knowledge, no extensive study has analyzed significant correlations among individual or combinations of these types of patient data and the improvement of multiclass problems as considered in this review. An extensive exploration analysis in this field would help to choose patient data suitable for the considered classification task. Following the study of Haenssle et al [35], it would further be of interest to note which type of patient data influences the clinician’s decision the most. Studies comparing the benefit of specific patient data integration in the artificial intelligence system versus the clinician’s decision and, therefore, pointing out the opportunities of human-algorithm integration systems should be the subject of future research.

### Conclusions

All 11 studies published so far indicate that the integration of patient data into CNN-based skin lesion classifiers may improve classification accuracy. The studies mainly used patient data that were routinely recorded (age, sex, and lesion location). Regarding the technical details, the main differences in the presented approaches occur in the fusing techniques. Further research should be dedicated to systematically evaluating the impact of incorporation of individual and combined patient data into CNN-based classifiers to show its benefit reproducibly and transparently and to pave the way for the translation of these combined classifiers into the clinic.

## Appendix

Multimedia Appendix 1 Relevant references for the overview of the patient data illustrated.

## Abbreviations

AK: actinic keratosis
BCC: basal cell carcinoma
BKL: benign keratosis-like lesion
BMS: Bristol Myers Squibb
CNN: convolutional neural network
MSD: Merck Sharp & Dohme
NV: melanocytic nevus
SCC: squamous cell carcinoma
SK: seborrheic keratosis
VASC: vascular lesion
