# Supplementary material for: Integrating Patient Data Into Skin Cancer Classification Using Convolutional Neural Networks: Systematic Review
Source: J Med Internet Res. 2021 Jul 2;23(7):e20708. doi: 10.2196/20708 (PMC8285747; doi:10.2196/20708)
Supplement: Multimedia Appendix 1 [file jmir_v23i7e20708_app1.docx]

**Supplementary table 1**. Overview and references of patient data illustrated in Figure 1*.*

| **Category** | **Patient data** | **Reference** |
| --- | --- | --- |
| History of UVR exposure | Sun exposure & related habits | [[1]](https://paperpile.com/c/kaA81R/O4854), [[2]](https://paperpile.com/c/kaA81R/vzMDj), [[3]](https://paperpile.com/c/kaA81R/FW3DV), [[4]](https://paperpile.com/c/kaA81R/RGRRU) |
|  | Sunburns | [[4]](https://paperpile.com/c/kaA81R/RGRRU) |
| Whole body nevi count | Congenital nevi | [[3]](https://paperpile.com/c/kaA81R/FW3DV) |
|  | Common nevi | [[3]](https://paperpile.com/c/kaA81R/FW3DV), [[5]](https://paperpile.com/c/kaA81R/eS6sB), [[6]](https://paperpile.com/c/kaA81R/884of) |
|  | Atypical nevi | [[3]](https://paperpile.com/c/kaA81R/FW3DV), [[5]](https://paperpile.com/c/kaA81R/eS6sB), |
| Personal & family medical history | Melanomas or other skin cancers | [[3]](https://paperpile.com/c/kaA81R/FW3DV), [[7]](https://paperpile.com/c/kaA81R/4RoQS) |
|  | Genetic test, e.g. immunosuppression, FAMMM | [[3]](https://paperpile.com/c/kaA81R/FW3DV) |
|  | Actinic keratosis | [[8]](https://paperpile.com/c/kaA81R/QZGZC), [[9]](https://paperpile.com/c/kaA81R/PdUUz) |
| Phenotypic | Skin type | [[3]](https://paperpile.com/c/kaA81R/FW3DV), [[6]](https://paperpile.com/c/kaA81R/884of) |
|  | Eye color | [[10]](https://paperpile.com/c/kaA81R/zj3h6),[[11]](https://paperpile.com/c/kaA81R/btREU), [[12]](https://paperpile.com/c/kaA81R/uEEt6) |
|  | Hair color | [[13]](https://paperpile.com/c/kaA81R/gJ6LL),[[14]](https://paperpile.com/c/kaA81R/DzeWl), [[15]](https://paperpile.com/c/kaA81R/iDZUO), [[16]](https://paperpile.com/c/kaA81R/dhPEi) |
| Lesion characteristics | Asymmetry, border irregularity, color & texture | [[17]](https://paperpile.com/c/kaA81R/4vPrn) |
|  | Size | [[18]](https://paperpile.com/c/kaA81R/DI1wH), [[19]](https://paperpile.com/c/kaA81R/AixZ6) |
|  | Anatomic site | [[18]](https://paperpile.com/c/kaA81R/DI1wH), [[19]](https://paperpile.com/c/kaA81R/AixZ6) |
|  | Elevation | [[18]](https://paperpile.com/c/kaA81R/DI1wH), [[19]](https://paperpile.com/c/kaA81R/AixZ6) |
|  | Symptoms (e.g. itching, bleeding, pain) | [[18]](https://paperpile.com/c/kaA81R/DI1wH), [[19]](https://paperpile.com/c/kaA81R/AixZ6) |
|  | Evolution | [[18]](https://paperpile.com/c/kaA81R/DI1wH), [[19]](https://paperpile.com/c/kaA81R/AixZ6) |
| Demographic | Age | [[20]](https://paperpile.com/c/kaA81R/oqpPr), [[21]](https://paperpile.com/c/kaA81R/BhzhG) |
|  | Sex | [[20]](https://paperpile.com/c/kaA81R/oqpPr), [[21]](https://paperpile.com/c/kaA81R/BhzhG) |
|  | Ethnicity | [[22]](https://paperpile.com/c/kaA81R/FR4GF) |
|  | Occupation | [[23]](https://paperpile.com/c/kaA81R/EqbwN) |
|  | Residency | [[22]](https://paperpile.com/c/kaA81R/FR4GF) |

References

1. [National Institutes of Health summary of the Consensus Development Conference on Sunlight, Ultraviolet Radiation, and the Skin. Bethesda, Maryland, May 8-10, 1989. Consensus Development Panel. J Am Acad Dermatol. 1991;24: 608–612.](http://paperpile.com/b/kaA81R/O4854)

2. [IARC Working Group on the Evaluation of Carcinogenic Risks to Humans, International Agency for Research on Cancer. Solar and Ultraviolet Radiation. World Health Organization; 1992.](http://paperpile.com/b/kaA81R/vzMDj)

3. [Leitlinienprogramm Onkologie (Deutsche Krebsgesellschaft, Deutsche Krebshilfe, AWMF). S3 Leitlinie Prävention Hautkrebs. AWMF Registernummer: 032/052OL; Available:](http://paperpile.com/b/kaA81R/FW3DV) <http://leitlinienprogrammonkologie.de/Leitlinien.7.0.html>

4. [krebsdaten. 17 Dec 2019 [cited 5 Apr 2020]. Available:](http://paperpile.com/b/kaA81R/RGRRU) <https://www.krebsdaten.de/Krebs/DE/Content/Krebsarten/Melanom/melanom_node.html>

5. [Gandini S, Sera F, Cattaruzza MS, Pasquini P, Abeni D, Boyle P, et al. Meta-analysis of risk factors for cutaneous melanoma: I. Common and atypical naevi. Eur J Cancer. 2005;41: 28–44.](http://paperpile.com/b/kaA81R/eS6sB)

6. [Bataille V, Bishop JA, Sasieni P, Swerdlow AJ, Pinney E, Griffiths K, et al. Risk of cutaneous melanoma in relation to the numbers, types and sites of naevi: a case-control study. Br J Cancer. 1996;73: 1605–1611.](http://paperpile.com/b/kaA81R/884of)

7. [Marcil I, Stern RS. Risk of developing a subsequent nonmelanoma skin cancer in patients with a history of nonmelanoma skin cancer: a critical review of the literature and meta-analysis. Arch Dermatol. 2000;136: 1524–1530.](http://paperpile.com/b/kaA81R/4RoQS)

8. [Salasche SJ. Epidemiology of actinic keratoses and squamous cell carcinoma. Journal of the American Academy of Dermatology. 2000. pp. S4–S7. doi:](http://paperpile.com/b/kaA81R/QZGZC)[10.1067/mjd.2000.103342](http://dx.doi.org/10.1067/mjd.2000.103342)

9. [Evans T, Boonchai W, Shanley S, Smyth I, Gillies S, Georgas K, et al. The spectrum ofpatched mutations in a collection of Australian basal cell carcinomas. Human Mutation. 2000. pp. 43–48. doi:](http://paperpile.com/b/kaA81R/PdUUz)[10.1002/1098-1004(200007)16:1<43::aid-humu8>3.0.co;2-7](http://dx.doi.org/10.1002/1098-1004(200007)16:1%3C43::aid-humu8%3E3.0.co;2-7)

10. [Lock-Andersen J, Drzewiecki KT, Wulf HC. Eye and hair colour, skin type and constitutive skin pigmentation as risk factors for basal cell carcinoma and cutaneous malignant melanoma. A Danish case-control study. Acta Derm Venereol. 1999;79: 74–80.](http://paperpile.com/b/kaA81R/zj3h6)

11. [Titus-Ernstoff L, Perry AE, Spencer SK, Gibson JJ, Cole BF, Ernstoff MS. Pigmentary characteristics and moles in relation to melanoma risk. Int J Cancer. 2005;116: 144–149.](http://paperpile.com/b/kaA81R/btREU)

12. [Veierød MB, Weiderpass E, Thörn M, Hansson J, Lund E, Armstrong B, et al. A prospective study of pigmentation, sun exposure, and risk of cutaneous malignant melanoma in women. J Natl Cancer Inst. 2003;95: 1530–1538.](http://paperpile.com/b/kaA81R/uEEt6)

13. [Lasithiotakis K, Krüger-Krasagakis S, Ioannidou D, Pediaditis I, Tosca A. Epidemiological differences for cutaneous melanoma in a relatively dark-skinned caucasian population with chronic sun exposure. European Journal of Cancer. 2004. pp. 2502–2507. doi:](http://paperpile.com/b/kaA81R/gJ6LL)[10.1016/j.ejca.2004.06.032](http://dx.doi.org/10.1016/j.ejca.2004.06.032)

14. [Fargnoli MC, Piccolo D, Altobelli E, Formicone F, Chimenti S, Peris K. Constitutional and environmental risk factors for cutaneous melanoma in an Italian population. A case–control study. Melanoma Research. 2004. pp. 151–157. doi:](http://paperpile.com/b/kaA81R/DzeWl)[10.1097/00008390-200404000-00013](http://dx.doi.org/10.1097/00008390-200404000-00013)

15. [Naldi L, Lorenzo Imberti G, Parazzini F, Gallus S, La Vecchia C. Pigmentary traits, modalities of sun reaction, history of sunburns, and melanocytic nevi as risk factors for cutaneous malignant melanoma in the Italian population: results of a collaborative case-control study. Cancer. 2000;88: 2703–2710.](http://paperpile.com/b/kaA81R/iDZUO)

16. [Ródenas JM, Delgado-Rodríguez M, Herranz MT, Tercedor J, Serrano S. Sun exposure, pigmentary traits, and risk of cutaneous malignant melanoma: a case-control study in a Mediterranean population. Cancer Causes and Control. 1996. pp. 275–283. doi:](http://paperpile.com/b/kaA81R/dhPEi)[10.1007/bf00051303](http://dx.doi.org/10.1007/bf00051303)

17. [Nachbar F, Stolz W, Merkle T, Cognetta AB, Vogt T, Landthaler M, et al. The ABCD rule of dermatoscopy. High prospective value in the diagnosis of doubtful melanocytic skin lesions. J Am Acad Dermatol. 1994;30: 551–559.](http://paperpile.com/b/kaA81R/4vPrn)

18. [Marzuka AG, Book SE. Basal cell carcinoma: pathogenesis, epidemiology, clinical features, diagnosis, histopathology, and management. Yale J Biol Med. 2015;88: 167–179.](http://paperpile.com/b/kaA81R/DI1wH)

19. [Gordon R. Skin cancer: an overview of epidemiology and risk factors. Semin Oncol Nurs. 2013;29: 160–169.](http://paperpile.com/b/kaA81R/AixZ6)

20. [Scrivener Y, Grosshans E, Cribier B. Variations of basal cell carcinomas according to gender, age, location and histopathological subtype. Br J Dermatol. 2002;147: 41–47.](http://paperpile.com/b/kaA81R/oqpPr)

21. [Krensel M, Petersen J, Mohr P, Weishaupt C, Augustin J, Schäfer I. Estimating prevalence and incidence of skin cancer in Germany. J Dtsch Dermatol Ges. 2019;17: 1239–1249.](http://paperpile.com/b/kaA81R/BhzhG)

22. [Augustin J, Kis A, Sorbe C, Schäfer I, Augustin M. Epidemiology of skin cancer in the German population: impact of socioeconomic and geographic factors. J Eur Acad Dermatol Venereol. 2018;32: 1906–1913.](http://paperpile.com/b/kaA81R/FR4GF)

23. [Pion IA, Rigel DS, Garfinkel L, Silverman MK, Kopf AW. Occupation and the risk of malignant melanoma. Cancer. 1995;75: 637–644.](http://paperpile.com/b/kaA81R/EqbwN)
